# Effects of a Dicalcium and Tetracalcium Phosphate-Based Desensitizer on *In Vitro* Dentin Permeability

**DOI:** 10.1371/journal.pone.0158400

**Published:** 2016-06-30

**Authors:** Jianfeng Zhou, Ayaka Chiba, Debora L. S. Scheffel, Josimeri Hebling, Kelli Agee, Li-na Niu, Franklin R. Tay, David H. Pashley

**Affiliations:** 1 Department of Prosthodontics, Peking University School and Hospital of Stomatology, Beijing, China; 2 Department of Cariology and Operative Dentistry, Graduate School of Medical and Dental Sciences, Tokyo Medical and Dental University, Tokyo, Japan; 3 Department of Orthodontics and Pediatric Dentistry, Universidade Estadual Paulista-UNESP, Araraquara Dental School, Araraquara, São Paulo, Brazil; 4 The Dental College of Georgia, Augusta University, Augusta, GA, United States of America; 5 State Key Laboratory of Military Stomatology & National Clinical Research Center for Oral Diseases & Shaanxi Key Laboratory of Oral Diseases, Department of Prosthodontics, School of Stomatology, The Fourth Military Medical University, Xi’an, China; University of Washington, UNITED STATES

## Abstract

The present study evaluated the effectiveness of a dicalcium and tetracalcium phosphate-based desensitizer in reducing dentin permeability *in vitro*. Dentin fluid flow was measured before and after treatment of dentin with patent dentinal tubules using 1 or 3 applications of the dicalcium and tetracalcium phosphate containing agent Teethmate^TM^ (TM) and comparing the results with two sodium fluoride varnishes Vella^TM^ (VLA) and Vanish^TM^ (VAN), after storage in artificial saliva for 24 h, 48 h and 7 days. Significant differences were observed among the 4 methods employed for reducing dentin permeability (p < 0.001) and the 3 post-treatment times (p < 0.001). VLA and VAN never achieved 50% permeability reductions consistently in any of the 3 time periods. Only the calcium phosphate-based desensitizer applied for 3 times consistently reduced dentin permeability by 50% after 24 h. When applied once, the permeability reduction of TM increased progressively over the 3 time periods. After 7 days, only one and three applications of the calcium phosphate-based desensitizer consistently reduced dentin permeability by more than 50%. Permeability reductions corresponded well with scanning electron microscopy examination of dentinal tubule orifice occlusion in dentin specimens treated with the agents. Overall, the dicalcium and tetracalcium phosphate-based desensitizer is effective in reducing dentin permeability via a tubule occlusion mechanism. The ability of the agent to reduce dentin permeability renders it to be potentially useful as a clinical dentin desensitizing agent, which has to be confirmed in future clinical studies. By contrast, the two sodium fluoride varnishes are not effective in dentin permeability reduction and should be considered as topical fluoride delivering agents rather than tubular orifice-blocking agents.

## Introduction

Cervical dentin hypersensitivity, the most common cause of reversible pulpitis of the dental pulp [1–3], is a common clinical problem encountered by adult patients [4,5]. Globally, this painful clinical condition affects 4–74% of the general population [6–20], more so in patients with periodontal diseases [21–23]. The condition is caused by the loss of enamel or cementum/gingival covering of root dentin. The exposed dentin with patent dentinal tubules reacts to thermal, osmotic, evaporative, or tactile stimuli by minute fluid shifts within dentinal tubules that activate A-δ pulpal nerve fiber endings and cause pain [24–28].

Reduction in dentin permeability involves the use of dentin tubule blocking agents such as potassium oxalate, calcium phosphates, arginine-calcium carbonate or dental adhesive materials [28–32]. Fluoride varnishes that are used for topical fluoride treatment have also been claimed to reduce dentin permeability [33–39]. Although fluoride ions do not contribute directly to tubular occlusion, acidic fluoride varnishes react with dentin to release calcium ions that can form CaF_2_ with the varnish base; the CaF_2_ particles can migrate into and occlude the dentinal tubules [40]. Penetration of the varnish base into the dentinal tubules, in turn, helps to impede dentinal fluid movement, and to release high concentrations of fluoride ions to produce intratubular calcium fluoride or fluoroapatite, thereby blocking the dentinal tubules [41].

Another dentinal tubule occluding agent combines tetracalcium phosphate (TTCP) and anhydrous dicalcium phosphate (DCPA) with a proprietary vehicle to create a paste that occludes open dentinal tubules within a clinically relevant time frame. The tubule occluding material [Teethmate^TM^ (TM), Kuraray Noritake Dental Inc., Tokyo, Japan] can transform into biological apatite within hours [42–44], following its topical application on dentin with patent dentinal tubules.

The objective of the present study was to evaluate the efficacy of the dicalcium and tetracalcium phosphate agent in reducing the permeability of human dentin *in vitro*, using a previously-established fluid filtration method. The results after exposure to artificial saliva for different time periods were compared to those derived from the use of two 5% sodium fluoride (NaF)-containing varnishes. The ability of these agents in occluding patent dentinal tubule orifices was also qualitatively investigated using scanning electron microscopy. The null hypothesis tested was that there are no differences in the ability of the dicalcium and tetracalcium phosphate cement and the two 5% NaF varnishes in reducing dentin permeability via a tubular occlusion mechanism.

## Materials and Methods

### Teeth and Dentin Preparation

Seventy-five human unerupted third molars were obtained following a protocol approved by the Human Assurance Committee of The Dental College of Georgia, Augusta University. Signed informed consent was received from each tooth donor. The teeth were stored frozen until use.

The teeth were thawed on the day of use. The occlusal enamel and superficial dentin were removed using a diamond-encrusted copper disk in an Isomet saw (Buehler Ltd., Lake Bluff, IL, USA), exposing a flat mid-coronal dentin surface. A second section was made 5 mm below the first, at right angle to the longitudinal axis of the tooth, creating a 5 mm-thick crown segment. After careful removal of the pulp tissues without touching the dentin walls, the pulp chamber was abundantly rinsed with deionized water. The flat dentin side (occlusal side) of the crown segments was polished with increasingly fine (320, 800, 1200, 2400 and 4000 grit) wet silicon carbide abrasive paper to remove the bulk of the smear layer. The thin remnant smear layer from each tooth was then etched with 0.5 M ethylenediaminetetraacetic acid (pH 7.4) for 2 min to render the dentinal tubules patent.

### Dentin Permeability

A hole slightly smaller than an 18 gauge stainless steel tube was drilled at the center of, and through a block of 2 x 2 x 1 cm thick polycarbonate to create a disposable dentin permeability stage. A 1.5 cm-length 18 gauge stainless steel tubing was forced from the bottom to the top of the block through the hole.

**Fig 1:**
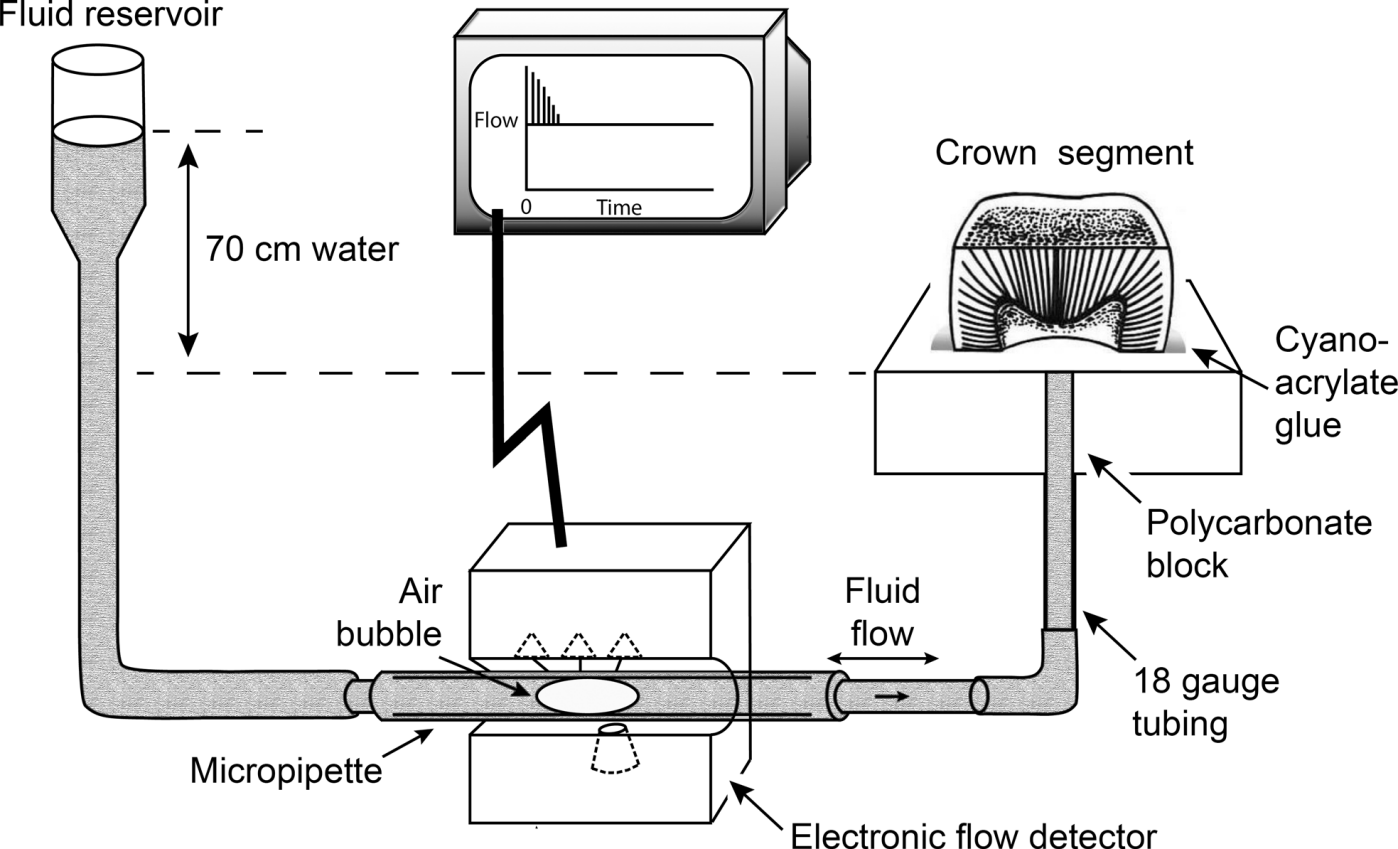
Schematic diagram of the dentin permeability testing setup.

Each crown segment was glued to the polycarbonate stage using a cyanoacrylate cement (Zapit Dental Ventures of America, Corona, CA, USA). A 25 gauge hypodermic needle was attached to a 3 cm^3^ syringe filled with deionized water. The hypodermic needle was inserted into the 18 gauge stainless steel tubing; deionized water was injected through the tubing to remove air beneath the crown segment (Fig 1).

The maximum fluid flow from each crown segment with patent dentinal tubules was measured with a Flodec device (DeMarco Engineering, Geneva, Switzerland) using 70 cm water pressure (6.86 kPa). This measurement was taken to be the pre-treatment baseline dentin permeability of the respective crown segment. Five, 2-min measurements were made to obtain the mean baseline permeability of each crown segment. Crown segments with baseline dentin permeability values between 2–5 μL min^-1^ were selected. Specimens with baseline dentin permeability higher or lower than the pre-determined range were excluded. Using this criterion, 56 out of the original 75 crown segments were used for the experiment.

Each crown segment served as its own control. That is, the pre-treatment (baseline) fluid flow was assigned a value of 100% permeability and the post-treatment permeability was expressed as a percent of that maximum value. Post-treatment dentin permeability was evaluated at 24 h, 48 h and 7 days after the application of the respective desensitizer.

### Experimental Design

The compositions of the desensitizer products are listed in Table 1. The twenty-eight crown segments were allotted to four groups (n = 14) with no statistically different baseline permeability (one-factor analysis of variance, p = 0.997). All materials were used according to the respective manufacturer’s instructions. Only the calcium phosphate-based desensitizer was applied for 1 and 3 times, as recommended by the manufacturer. All permeability measurements were performed by a single experienced examiner who was unaware of the material applied on each specimen.

**Table 1:** Brand name, manufacturer and major components of the dentin desensitizing products.

Brand name	Manufacturer	Major components	Batch
Teethmate^TM^ (TM)	Kuraray Noritake Dental Inc., Tokyo, Japan	*Powder*: Tetra-calcium phosphate, dicalcium phosphate anhydrous *Liquid*: Water, preservative	061114
Vanish^TM^ (VAN)	3M ESPE, St. Paul, MN, USA	5% NaF, tri-calcium phosphate	N689121
Vella^TM^ (VLA)	Preventive Technologies Inc., Indian Trial, NC, USA	5% NaF (22,600 ppm F^-^), xylitol	46794

#### Calcium phosphate desensitizer groups (TM; single or triple application)

After obtaining baseline dentin permeability values, one scoop of the calcium phosphate-based desensitizer powder was mixed with one drop of liquid for 20 sec. The slurry was the applied to the dentin surface for 40 sec using a microbrush with continuous rubbing movement. Care was taken to cover the entire dentin surface. Then, the slurry was rinsed off the dentin surface with deionized water for 2 sec.

For the triple application of the calcium phosphate-based desensitizer TM, the application steps described above were repeated for 3 times. After the last 2-sec rinse, each specimen was stored individually in a 20 mL glass vial containing artificial saliva to simulate exposure of the desensitizer-treated dentin to saliva in the oral cavity. The composition of the artificial saliva (in g/L, all ingredients obtained from MilliporeSigma, St. Louis, MN, USA; pH adjusted to 7.20 with KOH) was: sodium caboxymethyl cellulose (10), methyl-p-hydroxybenzoate (2), KCl (0.625), MgCl_2_·6H_2_O (0.059), CaCl_2_·2H_2_O (0.166), K_2_HPO_4_ (0.804) and KH_2_PO_4_ (0.326) [45]. The vials were kept in a 37°C incubator during the entire experiment, except during fluid filtration measurement. Each vial was removed from the incubator and allow to equilibrate with ambient temperature for 30 min prior to fluid filtration measurement.

#### 5% NaF white varnish with tri-calcium phosphate (VAN)

The content of an individual unit-dose VAN package (3M ESPE, St. Paul, MN, USA) was mixed well for 10 sec. A thin layer of the mixed varnish was applied evenly to the dentin surface of seven mounted specimens. Without rinsing, the surface of the treated crown segment was covered with artificial saliva, to enable the water component of the artificial saliva to harden the varnish, enabling the latter to remain on the dentin surface. The specimens were stored at 37°C in artificial saliva-containing vials, as described previously.

#### 5% NaF clear varnish with xylitol (VLA)

After blot-drying the dentin surface, one packet of VLA (Preventive Technologies, Inc. Indian Trial, NC, USA) was mixed in its own well for 10 sec, using the brush provided by the manufacturer. A thin layer of the mixed varnish was applied evenly to the dentin surface of seven mounted specimens and left undisturbed for 20 sec. Without rinsing, the varnish was covered with artificial saliva collected at the moment of varnish application. The specimens were stored at 37°C in artificial saliva-containing vials, as described previously.

#### Statistical analysis

Dentin permeability was calculated as a percentage of the baseline value, which was considered to be 100% permeability. Because the original data was not normally distributed, the percent values were non-linearly transformed to satisfy the normality and homoscedasticity assumptions for parametric statistical analysis. The transformed data was analyzed using two-factor repeated-measures analysis of variance (ANOVA) to examine the effects of desensitizer treatment (TM single application, TM triple application, VAN and VLA) and post-treatment time (24 h, 48 h and 7 days), and the interaction of those two factors on dentin permeability. Post-hoc pairwise comparison procedures were performed using the Holm-Šídák method. For all analyses, statistical significance was pre-set at α = 0.05.

### Scanning Electron Microscopy

Eighteen additional crown segments were prepared as described previously for scanning electron microscopy (SEM). Two specimens were used as the control. Because it was not feasible to reuse dehydrated specimens that had been examined with high-vacuum for SEM analysis, different desensitizer-treated specimens (N = 2) were used for evaluation after 24 h and 7 days of immersion in artificial saliva.

Both VLA and VAN produced a thin film of hardened varnish on the dentin surface. These films were carefully peeled off with a pair of surgical forceps. All specimens were then rinsed with deionized water for 10 sec, and kept overnight in closed containers containing dry calcium sulfate (Drierite, W.A. Hammond, Xenia, OH, USA). The dried specimens were mounted on aluminum stubs and sputter-coated with gold-palladium. Specimens were observed using a scanning electron microscope (XL30, FEI, Hillsboro, OR, USA) at 10–20 kV. Five thousand to 10,000x images were taken of each specimen at random locations. Following the acquisition of each low magnification image, a 30,000x image was taken to identify the extent of occlusion of the dentinal tubules.

## Results

### Reduction in Dentin Permeability

**Fig 2:**
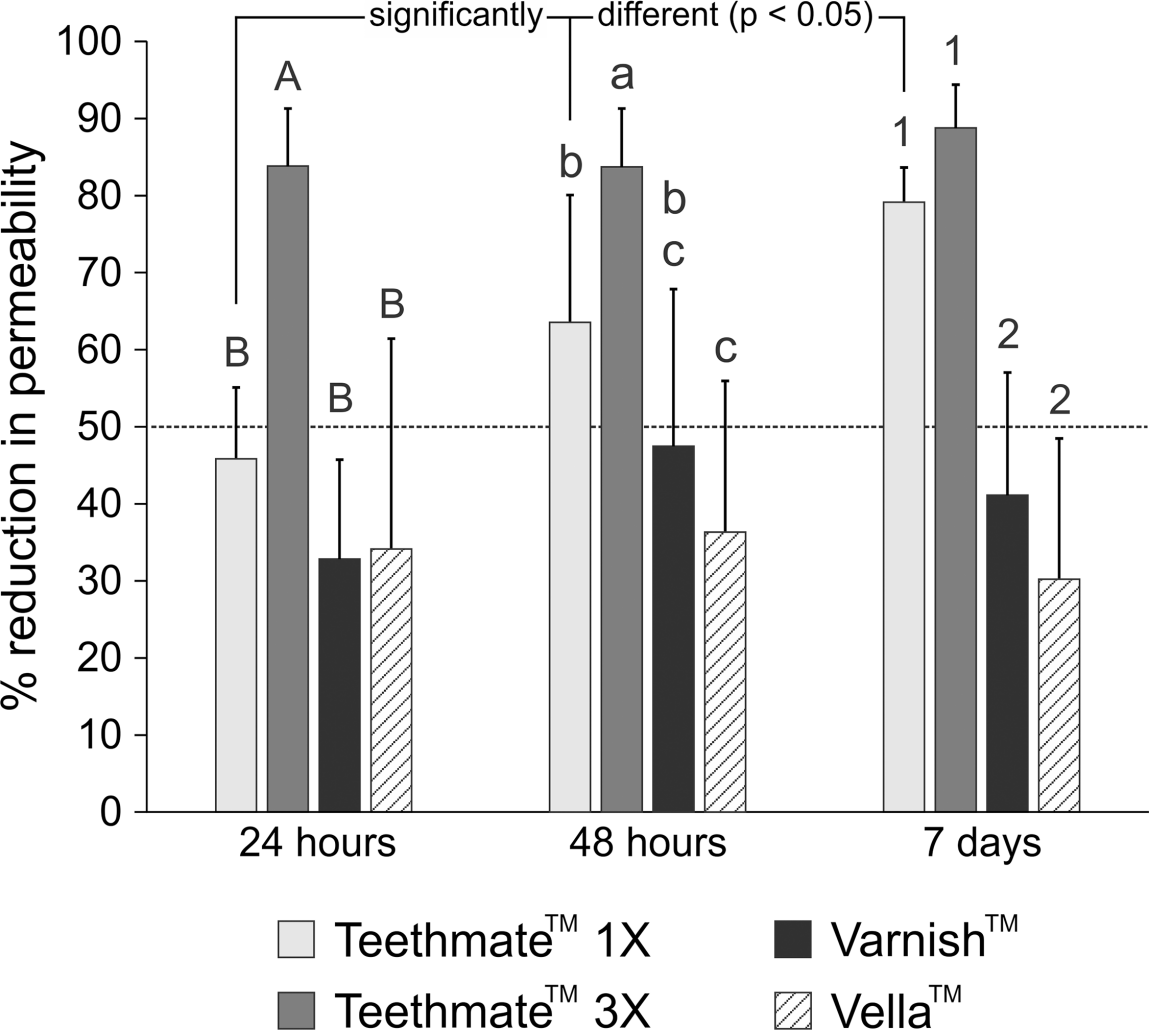
Reduction in dentin permeability, expressed as a percentage of the respective baseline permeability (100%) value of each individual dentin crown segment, after the application of 4 dentin desensitizers that had been aged for 3 time periods. Values represent means and standard deviations (n = 14). 1X: one application; 3X: three applications. For 24 h, desensitizer treatments labeled with the same upper case letters are not significantly different (P > 0.05). For 48 h, desensitizer treatments labeled with the same lower case letters are not significantly different (P > 0.05). For 7 days, desensitizer treatments labeled with the same numerical designators are not significantly different (P > 0.05). For time periods within each desensitizer treatment modality, pairwise comparisons of the three periods in the TM 1X group (linked with a black line) are all significantly different (P < 0.05). There are no significant differences for pairwise comparisons of time periods in the other three treatment groups.

After 24 h of exposure to artificial saliva, the percent reduction in dentin permeability of the crown segments (mean ± standard deviation, n = 14) for TM one application (1X), 3 applications, VAN and VLA were 45.8 ± 9.3%, 83.8 ± 7.5%, 32.8 ± 12.9% and 34.1 ± 27.3%, respectively (Fig 2). After 48 h, the percent reduction in dentin permeability of the same crown segments for TM 1X or 3X, VAN and VLA were 63.6 ± 16.5%, 83.7 ± 7.6%, 47.5 ± 20.4% and 36.3 ± 19.5%, respectively. When a 50% cut-off was applied to the data from these two time periods (Fig 2), only TM when applied 3X was capable of consistently reducing dentin permeability by 50%. After 7 days, the percent reduction in dentin permeability of the same crown segments for TM applied once *vs* 3X, VAN and VLA were 79.2 ± 4.5%, 88.8 ± 5.6%, 41.1 ± 15.9% and 30.2 ± 18.3%, respectively. For the 7-day period, only TM applied 1 or 3X was capable of consistently reducing dentin permeability by more than 50%, whereas VLA never reduced dentin permeability by more than 50%.

Two factor repeated-measures analysis of variance (Shapiro-Wilk test for normality: p = 0.204; modified Levene test for equal variance: p = 0.301) identified significance differences in percent permeability reductions among the 4 desensitizer treatments (p < 0.001) and the 3 post-treatment times (p < 0.001). The interaction of the two factors was also significant (p < 0.001). For multiple comparisons of the factor “desensitizer treatment”, all pairwise comparisons between treatment modalities were significantly different except for the comparison between VAN and VLA (p = 0.548). For the factor “time period”, all pairwise comparisons between artificial saliva immersion times were significantly different except for the comparison between 48 h and 7 days (p = 0.161).

After 24 h, pairwise comparisons of the 4 desensitizer treatment modalities were all significantly different (all p < 0.001) except for TM 1X *vs* VAN, VLA *vs* VAN and TM 1X *vs* VLA. After 48 h, comparisons were all significantly different (all p < 0.005) except for TM 1X *vs* VAN and VLA *vs* VAN. After 7 days, comparisons were all significantly different (all p < 0.001) except for TM 1X *vs* TM 3X and VLA *vs* VAN. For pairwise comparisons of “time periods” within “desensitizer treatment”, significant differences were only identified among the three time periods when TM was used (all p < 0.005). No significant differences in percent dentin permeability reduction could be identified between time periods in the other 3 desensitizer treatment modalities (Fig 2).

### Scanning Electron Microscopy

Examination of control specimens that had not been treated with any desensitizer confirmed the absence of a smear layer covering the dentin surface (not shown). Dentin tubule orifices were all rendered patent and were surrounded by a peripheral cuff of peritubular dentin. There were no exposed collagen fibrils in the intertubular dentin, indicating that the dentin matrix remained mineralized after treatment with the calcium chelating solution used for producing tubular patency

**Fig 3:**
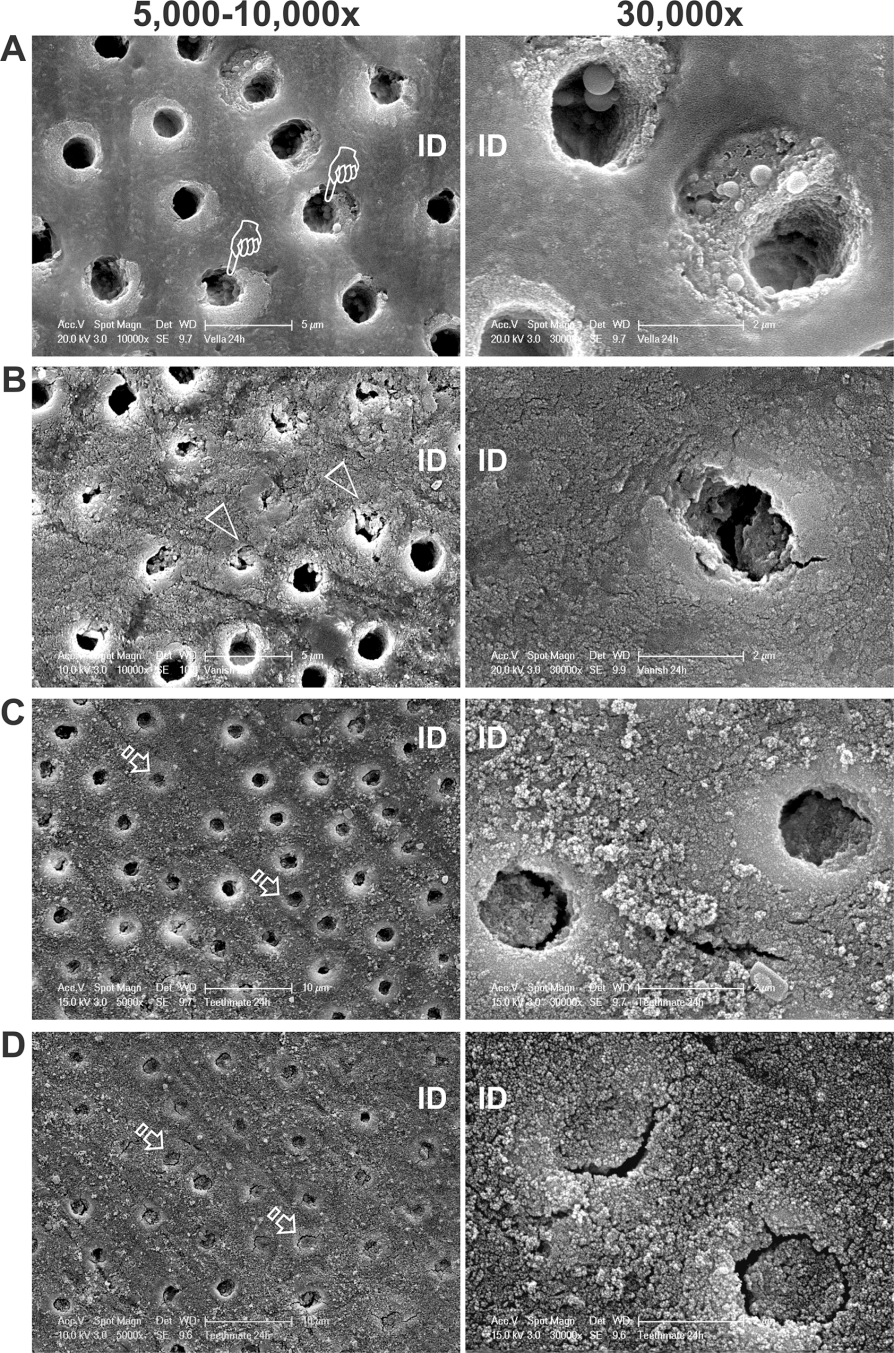
Scanning electron microscopy images (left: low magnification at 5,000–10,000x; right: high magnification at 30,000x) of the extent of tubular occlusion in human coronal dentin after 24 h of treatment with the four desensitizer treatments. Dentinal tubules had been rendered patent prior to desensitizer application, as confirmed with control dentin specimens without desensitizer application. ID: mineralized intertubular dentin. **A**. VLA. Pointers: tubules containing intratubular globular deposits. **B**. VAN. Open arrowheads: occluded dentinal tubules. **C**. One application of TM. Open arrows: completely occluded tubules. **D**. Three applications of TM. Open arrows: completely occluded tubules.

After 24 h of artificial saliva immersion, VLA-treated dentin showed globular-like deposits within the dentinal tubules and on the exposed peritubular dentin (Fig 3A). For VAN, partial or complete occlusion of some dentin tubules could be identified, while most of the tubules remained wide open (Fig 3B). Similarly, patent dentinal tubules could be identified among occluded tubules treated with TM 1X (Fig 3C). After triple applications of TM, most of the dentin tubules were completely occluded with mineral deposits (Fig 3D).

**Fig 4:**
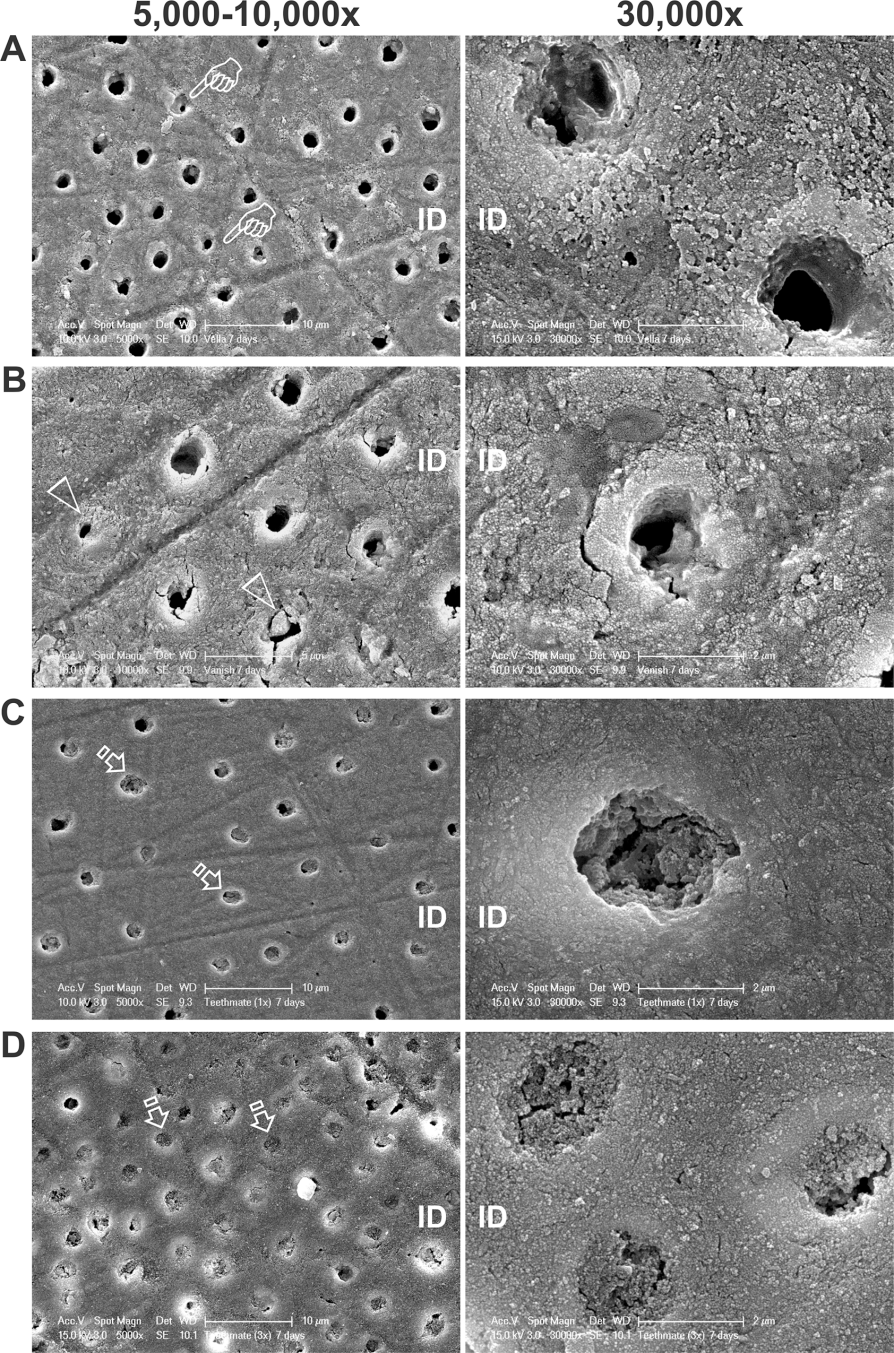
Scanning electron microscopy images (left: low magnification at 5,000–10,000x; right: high magnification at 30,000x) of the extent of tubular occlusion in human coronal dentin after 7 days of treatment with the four desensitizer treatments. ID: mineralized intertubular dentin. **A**. VLA. Pointers: partially-occluded dentinal tubules. **B**. VAN. Open arrowheads: partially-occluded tubules. **C**. One application of TM. Open arrows: completely occluded tubules. **D**. Three applications of TM. Open arrows: completely occluded tubules.

After 7 days, the VLA-treated specimens showed a completely different pattern from what was seen after 24 h. The initially observed intratubular globular deposits were no longer observed and dentinal tubules were predominantly patent (Fig 4A). Because the tubules were not examined by split fracture, no information could be obtained with respect to whether there were subsurface globular deposits within the dentinal tubules. The VAN-treated dentin showed similar tubular occlusion features as those observed after 24 h, with partial occlusion of some dentinal tubules; completely occluded tubules were rarely seen (Fig 4B). Dentin treated with a single application of TM had fewer patent tubules (Fig 4C) when compared with similarly-treated specimens examined after 24 h. Although some open tubules were seen after a single application of TM, patent tubules were rarely observed after three applications of the same desensitizer (Fig 4D). Intratubular deposits that occluded the tubular orifices were similar in appearance for both the 1X and 3X applications of TM.

## Discussion

If one attempts to desensitize sensitive dentin by tubule occlusion with inorganic crystals that are white, one is limited to various forms of calcium phosphates (Table 2), calcium carbonate, calcium hydroxide, calcium fluoride, or calcium oxalate. Perusal of Table 2 reveals that Ca(OH)_2_, calcium oxalate, calcium carbonate and calcium fluoride have solubility product constants (Ksp) that vary from 10^−6^ to 10^−10^. In contrast, the Ksp values of most calcium phosphate salts range from 10^−25^ to 10^−50^, with the exception of the Ksp for dicalcium phosphate dihydrate, which is only 10^−6.9^ [46,47].

**Table 2:** Solubility product constants (Ksp) at 25°C.

Insoluble calcium salts	Ksp
Calcium hydroxide Ca(OH)_2_	4.68 x 10^−6^
Di-calcium phosphate anhydrate CaHPO_4_	3.8 x 10^−6.9^
Calcium oxalate CaC_2_O_4_	1.42 x 10^−8^
Calcium carbonate CaCO_3_	4.68 x 10^−9^
Calcium fluoride CaF_2_	1.46 x 10^−10^
Amorphous calcium phosphate	1 x 10^−25^
Tri-calcium phosphate Ca_3_(PO_4_)_2_	2.7 x 10^−33^
Tetra-calcium phosphate Ca_4_(PO_4_)_2_O	3.1 x 10^−38^
Biologic apatite (calcium-deficient, carbonate-containing)	1 x 10^−50^

The more negative the Ksp exponent, the lower the solubility of the salt is. That is, CaF_2_, CaCO_3_, Ca(OH)_2_ and calcium oxalate precipitates on dentin should dissolve faster than would tri- or octacalcium phosphate precipitates. The latter calcium phosphate can hydrolyze to apatite. The Ksp for biologic apatite is only slightly more insoluble than is octacalcium phosphate.

One of the disadvantages of many of these insoluble salts is their pH sensitivity. At pHs < 5.5, plaque fluid becomes under-saturated with respect to calcium and trivalent phosphate ions [48], allowing hydrogen ions to protonate the PO_4_^3-^ groups in calcium phosphates, creating HPO_4_^-^ ions that overstrain the crystalline lattice and cause them to dissolve. The carbonate ion in calcium carbonate to be transformed into H_2_CO_3_↔H_2_O + CO_2_ also leads to solubilization of calcium carbonate. Calcium fluoride can become Ca^++^ + 2HF in the presence of acids, leading to its more rapid solubilization at acidic pH.

Although saliva is saturated in calcium and phosphate with respect to apatite [48], during ingestion of foods and drinks, oral fluids can become under-saturated, especially when eating or drinking acidic foods. Fermentation of glucose by biofilm organisms lowers plaque fluid pH enough to allow solubilization of apatite. However, the buffering action of salivary bicarbonates can buffer oral fluids back to pH 7.4, raising the concentration of trivalent phosphate necessary to recreate octacalcium phosphate that can hydrolyze to biologic apatite, during episodes of remineralization [49–51]. The limitations in the present study are that only short-term reduction in dentin permeability was examined; also only single application of the two fluoride-containing desensitizers was performed. Triple application of the two fluoride-containing desensitizers may have improved their effects. Examination on the dentin permeability with multiple applications of the dicalcium and tetracalcium phosphate-containing desensitizers and the other two fluoride containing-desensitizers after long-term chemical or mechanical challenge aging are required in future studies.

Based on the results of the present study, it appears that reductions in dentin permeability may be more effectively achieved with multiple applications of calcium salt-based desensitizers. Sensitive Pro-Relief™ (Colgate-Pamolive, New York City, NY, USA), a desensitizing dentifrice, contains CaCO_3_ for that purpose. MI Paste (GC Corp., Tokyo, Japan) contains casein phosphopeptides and amorphous calcium phosphate [52] that is marketed as a remineralizing toothpaste. Johnson & Johnson (New Bruncwick, NJ, USA) is marketing a slightly acidic potassium oxalate-containing Listerine™ mouthrinse [53] for daily use by people with dentin sensitivity in Europe. These relatively new desensitizing products acknowledge the difficulty in preventing solubilization of desensitizing occluding agents by recommending the products to be applied daily. Compared with consumer-accessible desensitizers, the professional versions of these desensitizing products contain higher concentrations of the substances that cannot be sold over the counter. The ultimate goal in reducing dentin hypersensitivity is to permanently occlude patent, sensitive tubules so that the outward seepage of dentinal fluid stops long enough for salivary minerals to mineralize those intratubular deposits.

## Conclusions

Within the limits of the present *in vitro* study, it may be concluded that both single and triple applications of Teethmate^TM^, a dentin desensitizer containing dicalcium and tetracalcium phosphates, are effective in reducing dentin permeability *in vitro* by occluding dentinal tubules. Of the high-concentrated fluoride-containing varnishes, both Vanish^TM^ and Vella^TM^ provided some reduction in dentin permeability, but this effect was inconsistent among the specimens examined. The ability of the dicalcium and tetracalcium phosphate containing agent to reduce dentin permeability renders it potentially useful as a clinical dentin desensitizing agent, which has to be confirmed in future clinical studies.
